# The transcriptional responsiveness of LKB1 to STAT-mediated signaling is differentially modulated by prolactin in human breast cancer cells

**DOI:** 10.1186/1471-2407-14-415

**Published:** 2014-06-09

**Authors:** Katja Linher-Melville, Gurmit Singh

**Affiliations:** 1 Department of Pathology and Molecular Medicine, McMaster University, Hamilton, Ontario, Canada

**Keywords:** Breast cancer, STAT3, STAT5, LKB1, Prolactin, Interleukin 6, Promoter, Transcriptional regulation

## Abstract

**Background:**

Liver kinase 1 (LKB1) is an important multi-tasking protein linked with metabolic signaling, also controlling polarity and cytoskeletal rearrangements in diverse cell types including cancer cells. Prolactin (PRL) and Signal transducer and activator of transcription (STAT) proteins have been associated with breast cancer progression. The current investigation examines the effect of PRL and STAT-mediated signaling on the transcriptional regulation of LKB1 expression in human breast cancer cells.

**Methods:**

MDA-MB-231, MCF-7, and T47D human breast cancer cells, and CHO-K1 cells transiently expressing the PRL receptor (long form), were treated with 100 ng/ml of PRL for 24 hours. A LKB1 promoter-luciferase construct and its truncations were used to assess transcriptional changes in response to specific siRNAs or inhibitors targeting Janus activated kinase 2 (JAK2), STAT3, and STAT5A. Real-time PCR and Western blotting were applied to quantify changes in mRNA and protein levels. Electrophoretic mobility shift (EMSA) and chromatin immunoprecipitation (ChIP) assays were used to examine STAT3 and STAT5A binding to the LKB1 promoter.

**Results:**

Consistent with increases in mRNA, the LKB1 promoter was up-regulated by PRL in MDA-MB-231 cells, a response that was lost upon distal promoter truncation. A putative GAS element that could provide a STAT binding site mapped to this region, and its mutation decreased PRL-responsiveness. PRL-mediated increases in promoter activity required signaling through STAT3 and STAT5A, also involving JAK2. Both STATs imparted basally repressive effects in MDA-MB-231 cells. PRL increased *in vivo* binding of STAT3, and more definitively, STAT5A, to the LKB1 promoter region containing the GAS site. In T47D cells, PRL down-regulated LKB1 transcriptional activity, an effect that was reversed upon culture in phenol red-free media. Interleukin 6, a cytokine activating STAT signaling in diverse cell types, also increased LKB1 mRNA levels and promoter activity in MDA-MB-231 cells.

**Conclusions:**

LKB1 is differentially regulated by PRL at the level of transcription in representative human breast cancer cells. Its promoter is targeted by STAT proteins, and the cellular estrogen receptor status may affect PRL-responsiveness. The hormonal and possibly cytokine-mediated control of LKB1 expression is particularly relevant in aggressive breast cancer cells, potentially promoting survival under energetically unfavorable conditions.

## Background

Prolactin (PRL) affects a range of physiological processes to maintain homeostasis, playing important roles in the mammary gland (reviewed in [1]) and influencing reproduction, maternal behavior, the immune system, osteogenesis, blood vessel development, ion transport, and metabolism, among other diverse functions (reviewed in [2-5]). PRL has been definitively associated with the onset and progression of human breast cancer by increasing cell proliferation (reviewed in [6-8]), and may contribute to metastasis by inducing the motility of human breast cancer cells [9]. The human PRL receptor (PRLR) is widely expressed in diverse tissues, and signaling through PRLR initiates activation of several intracellular pathways, the most well-characterized being the Janus activated kinase (JAK)/signal transducer and activator of transcription (STAT) pathway (reviewed in [3,10]). Some of the key events that occur in the normal mammary gland during pregnancy, lactation, and involution, as well as in adipocytes and during tumorigenesis in the breast, are regulated by STAT proteins [2-4,7,10]. The activation of cytokine receptors, including PRLR, in response to ligand binding typically results in phosphorylation and activation of JAK/STAT. STATs dimerize, translocate to the nucleus, and bind to specific recognition sequences in the promoter regions of select target genes, thereby activating or repressing transcription [11,12]. Seven mammalian STAT proteins have been identified. STAT2 is activated by α/β interferon, STAT4 by interleukin (IL)-12, and STAT6 by IL-4 to IL-13, while STAT1, STAT3, STAT5A, and STAT5B are activated by a range of stimuli, including PRL and IL-6 [13,14]. Targeting Jak2 may protect against the onset of mammary tumorigenesis in mice [15,16], and various STAT proteins have also been associated with breast cancer. In particular, STAT3 and STAT5 are generally thought to mediate opposite effects in mammary carcinoma cells [17]. Several negative regulators of JAK/STAT signaling have been identified that are induced differently in a cell type-dependent manner. STAT activation may upregulate the expression of members of the Suppressors of cytokine signalling (SOCS) family [18,19]. Other inhibitors include the phosphatase SHP-1 and Protein inhibitors of activated STAT (PIAS), which specifically targets STAT3 [20], providing another level of complexity in regulating JAK/STAT signal transduction.

A novel mechanism by which PRL may contribute to breast cancer progression is through its action on liver kinase 1 (LKB1). Acting either as a kinase or by changing its subcellular localization, LKB1 has been associated with proliferation, cell cycle arrest, apoptosis, polarity/motility, and energy metabolism (reviewed in [21]), and has been described as a tumor suppressor during pulmonary tumorigenesis [22]. However, it has also been suggested that LKB1 is required to protect cells from apoptosis during energy stress by initiating adenosine monophosphate-activated protein kinase (AMPK) signaling, leading to suppression of mTOR and the activation of ATP-producing pathways [23-25]. The LKB1-AMPK pathway has been described as a means to rescue cancer cells from metabolic collapse [21]. We have previously shown that PRL activates the AMPK pathway in an LKB1-dependent manner in specific human breast cancer cell lines, most notably MDA-MB-231 cells [26].

Little is currently known regarding how the expression of LKB1 is regulated. One means of repression is through promoter methylation [27,28], and the LKB1 promoter has been reported to be hypermethylated in colorectal carcinomas and testicular tumors, although out of 51 cancer cell lines analyzed *in vitro*, only one cervical carcinoma and three colorectal cell lines were methylated at the LKB1 locus, also corresponding to loss of expression [27]. Estrogen may be an important regulator, as multiple estrogen response elements (EREs) within the human LKB1 promoter region confer a repressive action in estrogen receptor (ER)-positive MCF-7 human breast cancer cells [29]. We have shown previously that levels of total LKB1 mRNA and protein increase in MDA-MB-231 cells cultured in the presence of PRL [26]. Similar to PRL-responsive promoters that contain potential STAT binding sites, such as those controlling expression of the β-casein [30,31], cyclin D1 [32,33], fatty acid synthase [34], and pyruvate dehydrogenase kinase (PDK4) genes [35], a putative STAT binding/interferon gamma-activated sequence (GAS) motif in the distal human LKB1 promoter region was identified by computational analysis. The presence of this putative site suggested that LKB1 transcriptional activity could be regulated by STAT proteins. Others have shown that PRL, through JAK2, induces binding of STAT5 to a distal GAS site in the cyclin D1 promoter, thereby enhancing promoter activity in Chinese hamster ovary (CHO-K1) cells transfected with the long form (LF) of PRLR [32]. In adipocytes, STAT5A binds to a putative STAT site in the PDK4 promoter in response to PRL stimulation [35]. In the current investigation, we aimed to investigate the importance of the GAS site in the distal human LKB1 promoter region, and the potential mechanisms underlying the responsiveness of LKB1 to PRL, in a representative triple-negative breast cancer cell line. Our findings demonstrate that changes in LKB1 expression are, at least in part, transcriptionally regulated by STAT3, as well as STAT5A. Identifying the mechanisms that underlie the regulation of LKB1 expression in different breast cancer cells may provide new insights into how this protein responds to different stimuli, including PRL or other cytokines such as IL-6.

## Methods

### Materials

Antibodies for total LKB1, total and phospho-JAK2, STAT3, STAT5, and ACC, and β-tubulin, β-catenin, and calnexin were obtained from Cell Signaling Technologies, Inc, and Actin was from MP Biochemicals. The human PRLR antibody was purchased from R&D Systems. Individual aliquots of recombinant human PRL (Cedarlane, Lot #608PRL01) or recombinant human IL-6 (R&D Systems) were prepared at a concentration of 100 μg/mL by reconstituting the lyophilates in sterile water or sterile PBS with 0.1% BSA, respectively, and stored at -20°C. The STAT3 pathway inhibitor (E)-3(6-bromopyridin-2-yl)-2-cyano-N-((S0-1-phenylethyl)acrylamide) (WP1066) (Sigma), STAT5 inhibitor (Calbiochem), and MEK1/2 inhibitor PD098059 (NEB) were reconstituted in DMSO, individual aliquots were stored at -20°C, and cells were pretreated with vehicle or an appropriate working concentration for 1 hr at 37°C in 5% CO_2_ prior to addition of PRL for 24 hr. Cells were pretreated with 5 μM of WP1066, a concentration that was experimentally determined to be effective at degrading JAK2 protein and blocking STAT3 phosphorylation in MDA-MB-231 cells. The STAT5 inhibitor was used to treat cells at a 50 μM final concentration (Calbiochem), whilePD098059 was used at 20 μM [32]. Cells were pretreated with 10 μg of Actinomycin D (Sigma) for 1 hr prior to culture in the presence of PRL for 24 hr.

### Plasmid constructs

The cloning of the full-length LKB1 construct from -1889/+1109 into pGL3-Basic (Promega) and construction of the LKB1Δ-1083 truncation reporter construct were described previously [29]. The pRL-TK Renilla luciferase construct was obtained from Dr. Julang Li (University of Guelph). Mutation of the GAS site (5’-TTCCAAGAA-3’) within the distal LKB1 promoter region at -1152 was accomplished using the Site-Directed Mutagenesis kit (Stratagene) and complementary mutant oligonucleotides corresponding to the sequence 5′-CCAGCATTATCTCCAGA*TTagtttAA*GTTGGGGTGTGAGCCAG-3′ (the GAS site is italics; mutated base pairs in lowercase letters). Mutations were confirmed by bi-directional sequencing. The human PRLR LF (1869 bp of the coding sequence, GeneBank Accession M31661.1, GI:190361) [36] was PCR amplified from cDNA derived from MDA-MB-231 cells using the primers PRLR-LF-FOR (5’-ATGAAGGAAAATGTGGCATCTGC-3’) and PRLR-LF-REV (5’-TCAGTGAAAGGAGTGTGTAAAACATG-3’), and the resulting product was confirmed by sequencing and expressed in pcDNA3.1.

### Cell culture and transient transfections

All human cell lines were used in accordance with institutional biosafety guidelines. MDA-MB-231 human breast cancer cells at low passage (less than 20 passages, ATCC #HTB-26) were maintained in DMEM supplemented with 10% FBS, and Chinese hamster ovary (CHO-K1) cells (ATCC #CCL-61) were cultured in DMEM/F12 containing 5% FBS and penicillin/streptomycin. T47D cells were maintained in RPMI-1640 with 10% FBS, in either media containing phenol red or without phenol red. For assays, cells were plated into 6-well tissue culture-treated plates (Falcon) at 2.5 × 10^5^ cells/well 24 hr prior to manipulation. Cells were transfected using Lipofectamine 2000 (Invitrogen) as described previously [29]. To assess viable cell proliferation, cells were counted using a haemocytometer and trypan blue staining.

### Reporter gene assays

Luciferase activity of cell lysates was determined as previously described [29] using the Dual Luciferase Assay (Promega) and a Berthold luminometer. Luciferase values were corrected for transfection efficiency by determining the ratio of firefly/Renilla luciferase activity and expressed as relative units. All data were normalized to untreated pGL3-Basic.

### siRNAs

Experimentally verified siRNAs for JAK2 (Hs_JAK2_7), STAT3 (Hs_STAT3_7), STAT5A (Hs_STAT5A_2), LKB1 (Hs_STK11_7), and a negative control (Ctrl_Control_1) were obtained from Qiagen. Transient transfections were carried out as described previously using Hiperfect reagent (Qiagen). MDA-MB-231 cells plated into 6-well plates at 1.25 × 10^5^ cells/well 3 hr prior to treatment with siRNAs [26,29].

### Real time PCR

cDNA was prepared and quantitative real time PCR was carried out using primers to amplify human LKB1 and the RNA polymerase II housekeeping genes, which were previously optimized [26]. Primers described by others [37,38], resulting in a 200 bp product, were used to quantify mRNA levels of the human PRLR LF. Relative mRNA levels were calculated using the 2^-[Δ][Δ]Ct^ method [39], and results are presented as fold changes relative to untreated controls.

### Western blotting

Total cell lysates were prepared as described previously [26,29]. 50 μg of protein was subjected to SDS-PAGE electrophoresis on 10% polyacrylamide gels and transferred onto PVDF membranes, which were blocked in non-fat dry milk, incubated in 1:1000 diluted primary antibody, followed by incubation with the appropriate anti-rabbit IgG horseradish peroxidise (HRP) secondary antibody (1:3000, Cell Signaling Technology). Signals were detected using the ECL Plus Western Blotting Detection System (Amersham Biosciences) and exposed to film. Stripped membranes were re-probed with primary anti-Actin antibody and anti-mouse IgG-HRP.

### Densitometry

Densitometric analyses of blots were performed using Image J analysis software. Values were expressed as a percent change over the control value and are represented as the mean ± SE of at least 3 independent experiments. For total and phosphorylated proteins, values were corrected relative to actin and relative to total protein/actin, respectively.

### Co-Immunoprecipitation

Following various treatments, cells were lysed in 1X lysis buffer supplemented with protease inhibitors. 100 μg of non-sonicated, cleared lysate in a final volume of 200 μl (following a protocol provided by Cell Signaling Technology) were incubated with 2 μl of antibody against total JAK2 overnight at 4°C with end-over-end rotation, followed by the addition of 20 μl of protein A/G agarose (Invitrogen) and further incubation at 4°C for 3 hr. Samples were washed 5 times with lysis buffer prior to adding 4X SDS-sample buffer and boiling. The signal was detected following Western blotting with anti-JAK2 or anti-phospho-JAK2 primary antibodies and incubation with anti-rabbit IgG-HRP. As a negative control, normal rabbit IgG (SC-2027; Santa Cruz Biotechnology, Inc.) was used instead of specific antibody in one IP for each group of cells. A positive control was included during Western blotting, referred to as input, which represented 10% of cleared lysate.

### Preparation of nuclear extracts

Cells were cultured in 10-cm dishes in the absence and presence of 100 ng/mL of PRL for 24 hr before harvesting nuclear extracts using the NE-PER Cytoplasmic and Nuclear Extraction Reagents kit (Pierce) following the manufacturer’s protocol. Protein concentrations of nuclear extracts were determined using a Bradford assay.

### EMSA

Probe preparation and EMSAs were performed as previously described [40] using the DNA 3’ End Biotinylation kit (Pierce) and the LightShift Chemiluminescent EMSA kit (Pierce). EMSA probes consisted of biotinylated double-stranded oligonucleotides. Probe sequences are listed in Table 1, with the GAS and GASmut sequences in bold italics. For competitor assays, 200-fold molar excess of unlabeled, double-stranded probe, corresponding to 4 pmol, was included in EMSA reactions.

**Table 1:** EMSA probes

**Probe**	**Sequence (5’-3’)**	**Length**
**GAS**	AGCATTATCTCCAGA ***TACCAAGGG*** GTTGGGGTGTGAGCCA	40 bp
**GASmut**	AGCATTATCTCCAGA ***TTAGTTTAA*** GTTGGGGTGTGAGCCA	40 bp
**Oct1 (non-specific)**	AGAGGATCCATGCAAATGGACGTACG	26 bp

### ChIP assays

ChIP assays were carried out using the ChIP-IT Express Enzymatic kit (Active Motif) using a dounce homoginizer to lyse cells. Optimal enzymatic digestion of chromatin from MDA-MB-231 cells was empirically determined to occur after 10 min, yielding sheared chromatin that migrated between 200 and 1500 bp on an agarose gel. Equal DNA concentrations corresponding to 1.5 μg were applied to each set of immunoprecipitation reactions, which included either normal rabbit IgG, STAT3, or STAT5A antibody (sc-2027, sc-7179X, or sc-1081X, respectively; Santa Cruz Biotechnology). Samples were incubated with magnetic beads overnight at 4°C with end-over-end rotation. After reversal of cross-links, DNA precipitation, and clean-up, enriched DNA and input were analyzed by quantitative real time PCR with primers spanning the predicted GAS site, as well as primers specific to a region of the LKB1 promoter that does not contain a putative STAT binding motif (Table 2). The efficiency of each primer set was tested by producing a standard curve from two-fold dilutions of input, and the integrity of products was verified by agarose gel electrophoresis. Fold enrichment relative to IgG was calculated for immunoprecipitated samples, and data are presented normalized to values obtained for the negative binding region.

**Table 2:** Primers for ChIP

**Probe**	**Sequence (5’-3’)**	**Product size**
**LKB1-GAS-FOR**	GGACCTACCGATGCCAATTA	184 bp
**LKB1-GAS-REV**	TGGGCAATAAGAGCGAAACT	
**LKB1-Neg-FOR**	GAGGACGAAGTTGACCCTGA	208 bp
**LKB1-Neg-REV**	CAACAAAAACCCCAAAAGGA	

### Statistical analyses

Results represent the mean ± SEM of at least three independent replicates, and were analyzed by t-test (denoted by stars) or 1-way ANOVA with a Tukey’s post-test (denoted by different letters) to assess statistical differences between groups using GraphPad Prism software. Results were considered significant at p <0.05. For qualitative assays, including Western blots and EMSAs, the results shown are representative of at least two independent experiments.

## Results

### LKB1 plays an important role in MDA-MB-231 human breast cancer cells

**Figure 1:**
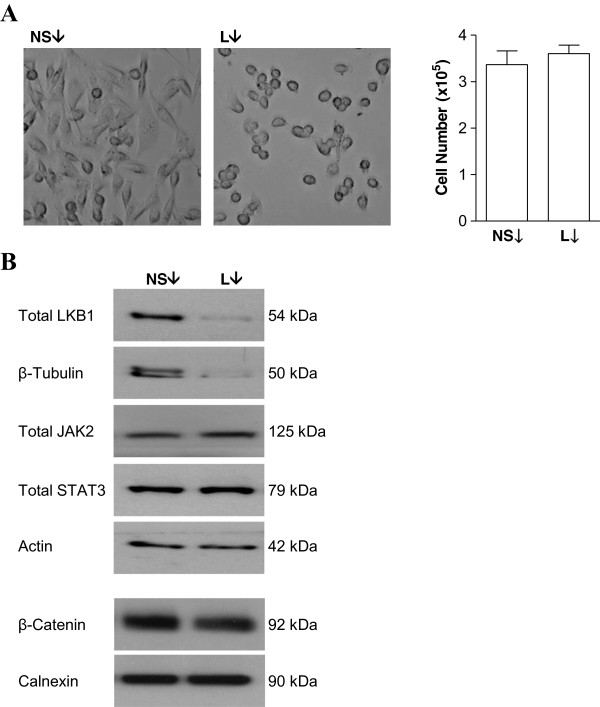
**LKB1 is functionally important in MDA-MB-231 human breast cancer cells. (A)** Knock-down of LKB1 using a specific siRNA in MDA-MB-231 cells results in distinct morphological changes without affecting the total number of viable cells compared to cells treated with a non-specific (NS) siRNA. 10X magnification of live cells using a Leica DMIL microscope. **(B)** A representative Western blot demonstrating that loss of LKB1 reduces β-tubulin and β-catenin protein levels without affecting the expression of other proteins.

We previously showed that LKB1 contributes to AMPK pathway activation in human breast cancer cells [26]. In the current study, we demonstrated that, beyond modulating cellular metabolism, LKB1 may also be important in regulating cell morphology. When cultured in DMEM supplemented with 10% FBS, untreated MDA-MB-231 cells display two distinct cell types, one spindle-shaped and the other more rounded. Knocking down LKB1 resulted in distinct morphological changes, with cells becoming more rounded compared to cells treated with a non-specific negative control siRNA (Figure 1A). Cell number or viability, which was assessed by trypan blue exclusion, were not affected (Figure 1A). LKB1 is known to affect cell polarity and motility, and interestingly, its knock-down resulted in decreased expression of β-tubulin, an important component of the cytoskeleton, at the protein level, without affecting the expression of other proteins, including actin and calnexin (Figure 1B). In addition, levels of β catenin, an epithelial marker that has also been implicated in WNT signaling, were also decreased (Figure 1B). It appears that LKB1 regulates several important cellular processes in human breast cancer cells, warranting further investigation into how its expression is controlled.

### MDA-MB-231 cells express the PRLR and are responsive to PRL

**Figure 2:**
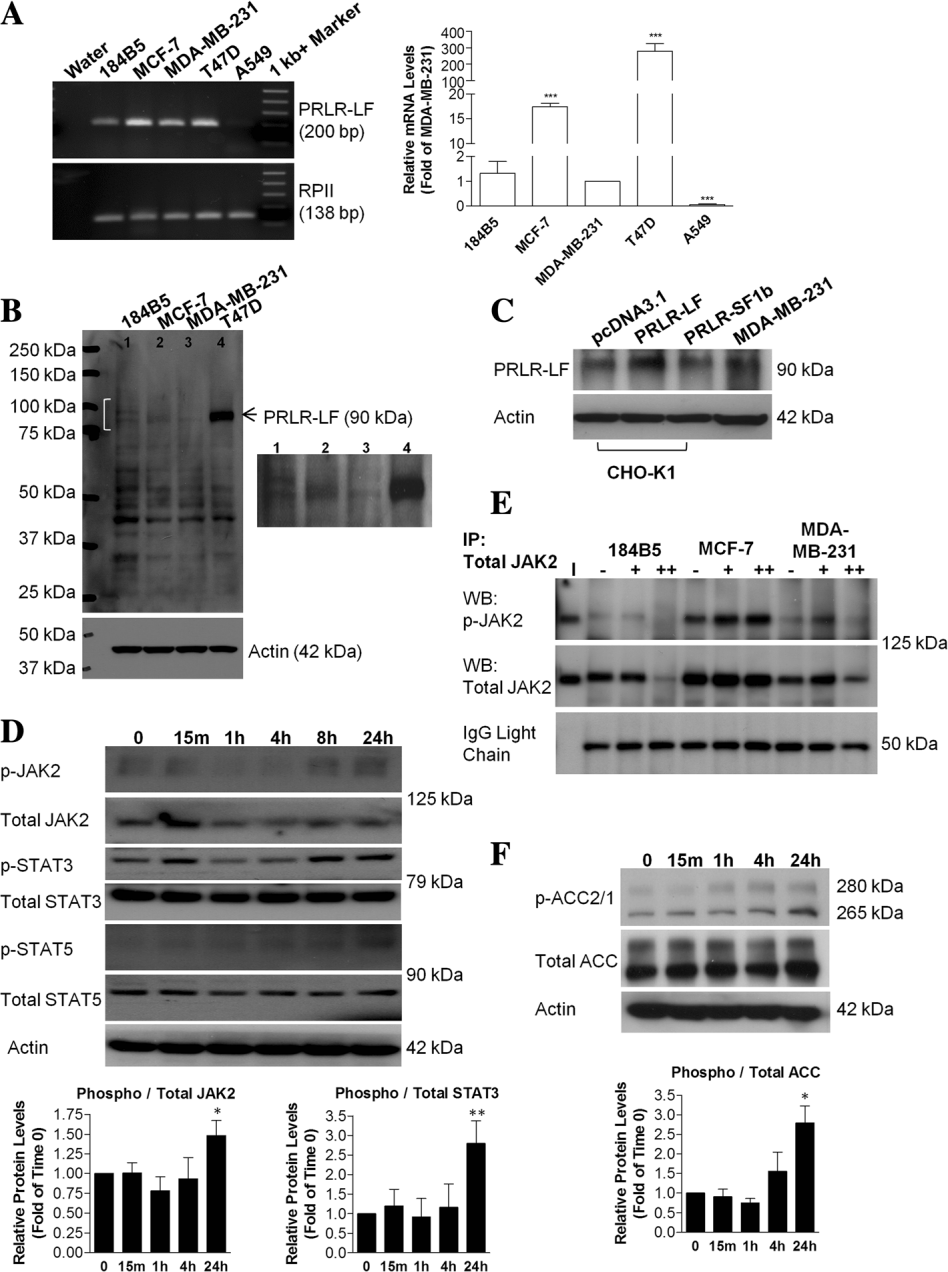
**PRL has the potential to directly signal to LKB1 in MDA-MB-231 cells. (A)** The PRLR LF is expressed at the mRNA level in representative breast cancer cells including MDA-MB-231 cells and 184B5 normal breast epithelial cells, while levels are close to undetectable in A549 lung cancer cells, as assessed by quantitative real time PCR. **(B)** Various isoforms of the PRLR are potentially expressed at the protein level in 184B5, MCF-7, and MDA-MB-231 cells. The LF migrates at the expected molecular weight of 85-90 kDa, similar to the band obtained in T47D cells, which express high levels of the LF, and **(C)** is comparable to migration in CHO-K1 cells transiently transfected with an expression vector encoding the LF of PRLR. **(D)** Representative Western blots of a time-course demonstrating that JAK2, STAT3, and STAT5 are phosphorylated in MDA-MB-231 cells cultured with 100 ng/mL of PRL for 24 hr. **(E)** Co-immunoprecipitations (IPs) were carried out using equal amounts of total cell lysates followed by Western blotting (WB). IPs with total JAK2 followed by WB with phospho- and total JAK2 were performed on lysates from 184B5, MCF-7, and MDA-MB-231 cells. I: 10% of total non-IP lysate or “input” as a positive control, -: no treatment, +: treated with 100 ng/mL of PRL for 24 hr, ++: pre-treated with 5 μM WP1066 for 2 hr followed by the addition of PRL for 24 hr. **(F)** PRL also temporally induced inactivation (phosphorylation) of ACC.

Our previous work demonstrated that PRL activates LKB1-AMPK-ACC signaling in MDA-MB-231 cells. PRL elicits cellular responses through the PRLR, with different receptor isoforms sharing common extracellular ligand binding and transmembrane domains, differing only in their intracellular regions due to alternative splicing. In humans, the known PRLR isoforms include the LF, as well as the delta S1, intermediate, and short forms (ΔS1, IF, SF1a and SF1b, respectively) and the PRLR binding protein (reviewed in [10]). We verified that PRL has the potential to directly signal through the PRLR in MDA-MB-231 cells by examining receptor mRNA and protein levels using T47D cells as a positive control for high expression of the LF. PRLR LF mRNA was detected in MDA-MB-231 cells (Figure 2A), consistent with reports by others [37,38]. Its expression at the protein level was assessed using the monoclonal anti-human PRLR antibody, which specifically recognizes the extracellular domain common to all known isoforms (R&D Systems, Inc.). Differences in mRNA levels were reflected at the protein level, with the LF migrating at approximately 85–90 kDa (Figure 2B). Additional bands were also present, which could either be non-specifics or other PRLR isoforms. It is possible that breast cancer cells could also express ΔS1, IF, SF1a, SF1b, or PRLRBP, as bands that correspond to their expected molecular weights were detected at 70, 50, 56, 42, and 32 kDa, respectively. To confirm the functional presence of PRLR in MDA-MB-231 cells, we compared protein levels to exogenously introduced PRLR LF expression in CHO-K1 cells, which exhibit low levels of endogenous PRLR (reviewed in [10])*.* Transient transfection of CHO-K1s with a mammalian expression vector encoding the full-length coding sequence of the human PRLR LF resulted in an approximately 2-fold increase in receptor levels compared to cells transfected with either empty vector (pcDNA3.1) or PRLR-SF1b encoding a short isoform (Figure 2C). Bands for the LF were detected at 85–90 kDa, consistent with migration of the endogenous band present at a similar molecular weight in MDA-MB-231 cells (Figure 2C).

We next examined potential signaling through STATs, as these proteins are commonly activated in response to PRL stimulation in cells that express the PRLR. A time course revealed that PRL induces a gradual increase in JAK2 and STAT3 phosphorylation in MDA-MB-231 cells in the presence of 100 ng/mL of PRL (Figure 2D). Densitometric analysis revealed that at 24 hr, the presence of PRL in the culture media increased phospho-JAK2 levels by 1.5-fold (p < 0.02) and phospho-STAT3 levels by 2.8-fold (p < 0.01) relative to time 0 (Figure 2D). An increase in phospho-STAT5 levels also occurred in response to PRL in MDA-MB-231 cells, although levels were very low. To confirm the phosphorylation of JAK2, we performed an immunoprecipitation (IP) for total JAK2 on lysates derived from 184B5, MCF-7, and MDA-MB-231 cells treated without and with PRL for 24 hr, or pretreated with WP1066, a drug that degrades total JAK2 protein, followed by Western blotting to detect both phospho- and total JAK2 (Figure 2E). IP of JAK2 in MDA-MB-231 cells confirmed its increased activation in the presence of PRL. Consistent with our previous findings [26], PRL inactivated ACC, temporally increasing its phosphorylation by 2.8-fold at 24 hr (p < 0.02) (Figure 2F).

### The LKB1 promoter is a target for PRL-mediated signaling

**Figure 3:**
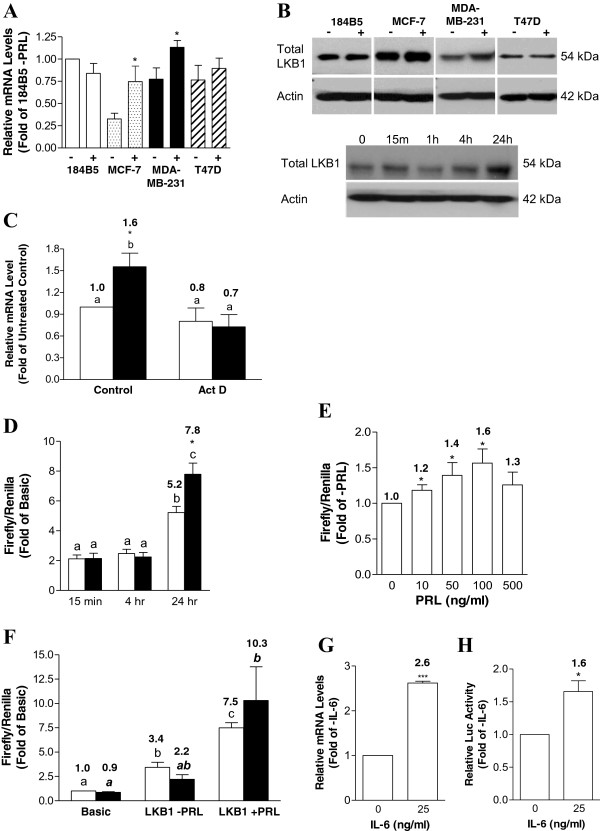
**PRL stimulates LKB1 promoter activity in MDA-MB-231 cells. (A)** PRL significantly increases LKB1 mRNA levels in MDA-MB-231 and MCF-7 cells. **(B)** Upper panel: a representative Western blot depicting LKB1 protein levels in 184B5, MCF-7, MDA-MB-231, and T47D cells cultured without and with 100 ng/mL of PRL for 24 hr. Lower panel: In MDA-MB-231 cells, LKB1 protein levels increase temporally in the presence of 100 ng/mL of PRL. **(C)** Pretreatment of MDA-MB-231 cells with Actinomycin D (Act D) for 1 hr abrogates PRL-mediated increases in LKB1 mRNA levels. Cells were untreated (open bars) or cultured with (black bars) 100 ng/mL of PRL for 24 hr. **(D)** Cells co-transfected with pGL3-Basic (Basic) or the full-length LKB1 reporter construct (LKB1) and pRL-TK were cultured without (open bars) or with (solid bars) 100 ng/mL of PRL for 15 min, 4 hr, or 24 hr. Lysates assayed for dual luciferase activity demonstrated a significant PRL-mediated increase at 24 hr. **(E)** PRL dose-dependently increased LKB1 promoter activity. Lysates from MDA-MB-231 cells co-transfected with LKB1 and pRL-TK and cultured without or with varying concentrations of PRL (10 to 500 ng/mL) for 24 hr were assayed for dual luciferase activity. **(F)** Cells treated with non-specific (open bars) or LKB1 (solid bars) siRNA for 48 hr were transfected with luciferase vectors and cultured without or with 100 ng/mL of PRL for 24 hr. Culture of MDA-MB-231 cells for 24 hr in the presence of 25 ng/mL of recombinant human IL-6 significantly increased **(G)** LKB1 mRNA levels and **(H)** LKB1 promoter activity in cells transfected with luciferase vectors. Data represent the mean of at least three independent experiments (±SEM) relative to controls, with different letters denoting significant differences between groups and a * indicating significant increases between the – and + PRL groups at 24 hr (p<0.05).

We have shown previously that PRL is able to up-regulate LKB1 protein levels in MDA-MB-231 cells [26]. A significant increase in LKB1 expression at the mRNA level was observed in MCF-7 and MDA-MB-231 cells following sustained PRL treatment, although no changes were observed in 184B5 normal breast epithelial cells, and only a very minor increase occurred in T47D cells (Figure 3A). These changes were reflected at the protein level (Figure 3B), and a time course in MDA-MB-231 cells revealed that maximal increases in LKB1 protein levels occurred after a 24 hr culture in the presence of PRL (Figure 3B). We therefore examined the potential involvement of PRL in regulating LKB1 expression at the transcriptional level. As shown in Figure 3C, 100 ng/mL of PRL significantly increased LKB1 mRNA levels by approximately 1.5-fold relative to the untreated control in MDA-MB-231 cells (p < 0.01), consistent with results in Figure 3A, while pretreatment with Actinomycin D completely abolished this effect. The transcriptional regulation of LKB1 by PRL was examined further using a human LKB1 promoter reporter construct, which included the regulatory region spanning -1889 to +1109 cloned upstream of a firefly luciferase gene [29]. A time course revealed that cotransfection of MDA-MB-231 cells with the full-length LKB1 promoter construct significantly increased luciferase activity by approximately 1.5-fold (p < 0.02) after a 24 hr culture in the presence of 100 ng/mL of PRL (Figure 3D). The effect on LKB1 promoter activity was dose-dependent, with a maximal 1.6-fold stimulation obtained using 100 ng/mL of PRL for 24 hr (p < 0.05; Figure 3E). Treatment with PRL also increased LKB1 transcriptional activity in MDA-MB-231 cells in which LKB1 was knocked down using a specific siRNA (Figure 3F), consistent with our previous findings [26]. In addition to PRL, we also examined the responsiveness of the LKB1 promoter to IL-6, which is also able to activate JAK/STAT signaling. Treating MDA-MB-231 cells with 25 ng/ml of recombinant human IL-6 for 24 hr significantly increased LKB1 mRNA levels by 2.6-fold (p < 0.001; Figure 3G), also significantly increasing promoter activity by 1.7-fold (p < 0.02; Figure 3H).

**Figure 4:**
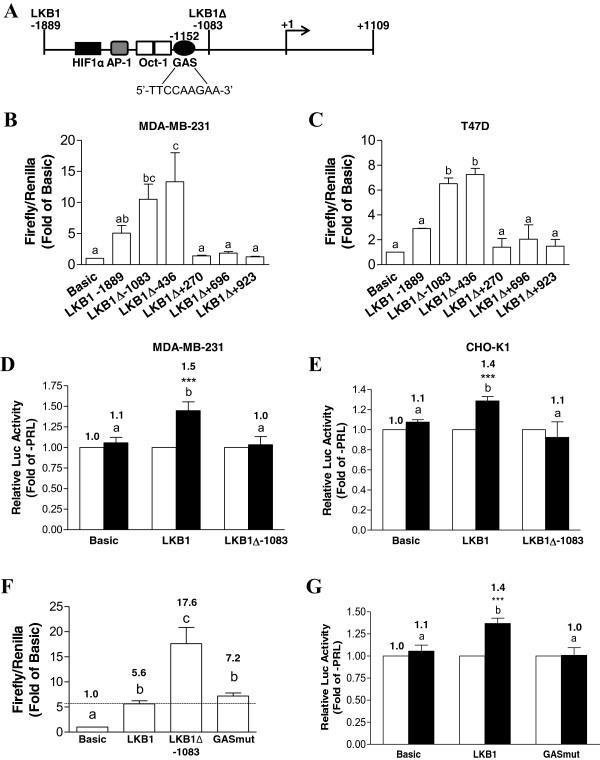
**Truncating a region from -1889 to -1083 or mutating a distal GAS site abrogate PRL-responsiveness of the LKB1 promoter. (A)** A diagrammatic representation of the human LKB1 promoter from -1889 to +1109 bp. A GAS consensus site (TTCCAAGAA), which may potentially be bound by STAT proteins, is located at -1152. In addition, putative binding sites for HIF1α (-1562), AP-1 (-1233), and OCT-1 (-1183, -1165) are indicated. The location of the LKB1Δ-1083 truncation is also shown. **(B)** MDA-MB-231 or **(C)** T47D cells were transiently co-transfected with either Basic, LKB1, or various promoter-luciferase truncation constructs (LKB1Δ-1083, -436, +270, +696, or +923) and pRL-TK and assayed for dual luciferase activity. **(D)** MDA-MB-231 cells were co-transfected with either LKB1 or LKB1Δ-1083 and pRL-TK, while **(E)** CHO-K1 cells were co-transfected with the PRLR LF, in addition to the constructs listed in (D), and both cell types were cultured without (open bars) or with (solid bars) 100 ng/mL of PRL for 24 hr before measuring dual luciferase activity. Data are presented relative to untreated controls. **(F)** MDA-MB-231 cells were co-transfected with LKB1, LKB1Δ-1083, or the LKB1 promoter-luciferase construct containing a mutated GAS site (GASmut) and pRL-TK, and lysates were assayed for dual luciferase activity. Data is presented relative to Basic. **(G)** Transfected cells were cultured without (open bars) or with (solid bars) 100 ng/mL of PRL for 24 hr before measuring dual luciferase activity, which is presented relative to the –PRL group. Data represent the mean of at least three independent experiments (±SEM). Different letters denote significant differences between groups (p<0.05), while a star (*) indicates statistically significant increases in PRL-treated LKB1 promoter activity compared to untreated controls.

Computational analysis using NSITE software (Softberry Inc.) revealed that, in addition to several EREs that we previously characterized in MCF-7 cells [29], the LKB1 promoter also contains a putative STAT/consensus GAS binding site (TTCNNNGAA) at -1152 bp, as well as a hypoxia-inducible factor 1 alpha (HIF1α), an activator protein 1 (AP-1), and two octamer-binding transcription factor 1 (OCT-1) sites (Figure 4A). The distal GAS site was of particular interest, given that PRL and cytokine stimulation are known to involve the activation and nuclear translocation of STATs, and STAT proteins mediate the action of cytokines at similar sites in other systems. Most STATs bind to consensus GAS sites, TTCN_m_GAA, where m = 4 for STAT6 and m = 3 for the optimal binding of all other STATs [41,42]. The sequence of the putative GAS site present in the LKB1 promoter, when reverse complemented, was found to be identical to both a PRL-responsive distal GAS site located in the human cyclin D1 promoter (TTCTTGGAA) [32,33] and a canonical STAT5 binding site (PRE) within the β-casein promoter [30,31], differing by only one base pair from a binding site described for STAT3 (TTCTGGGAA) [43]. Truncation analysis of the promoter region in MDA-MB-231 cells revealed the presence of a potential silencer element in the region spanning -1889 to -1083, as loss of this 800 bp fragment led to a significant 2-fold increase in promoter activity (Figure 4B), consistent with our previous findings reported in MCF-7 cells [29] and results obtained in T47D cells (Figure 4C). PRL-responsiveness was lost in MDA-MB-231 cells transiently transfected with LKB1Δ-1083, a truncated luciferase reporter construct lacking the putative GAS site (Figure 4D). As shown in Figure 4E, in CHO-K1 cells transiently co-transfected with the PRLR LF and the full-length LKB1 luciferase construct, 100 ng/mL of PRL significantly increased promoter activity by 1.4-fold (p < 0.0005), which was also lost when the promoter was truncated. The putative GAS site in the distal LKB1 promoter region was mutated to assess its contribution to the stimulatory effect of PRL on transcriptional activity in MDA-MB-231 cells. Compared to the significant increase on basal LKB1 promoter activity obtained using LKB1Δ-1083, mutation of the GAS site had only a mild repressive effect, a change that was not statistically significant (Figure 4F). Importantly, the LKB1 full-length promoter with the mutated GAS site did not respond to PRL (Figure 4G).

### STAT signaling is important for basal and PRL-mediated activation of the LKB1 promoter

**Figure 5:**
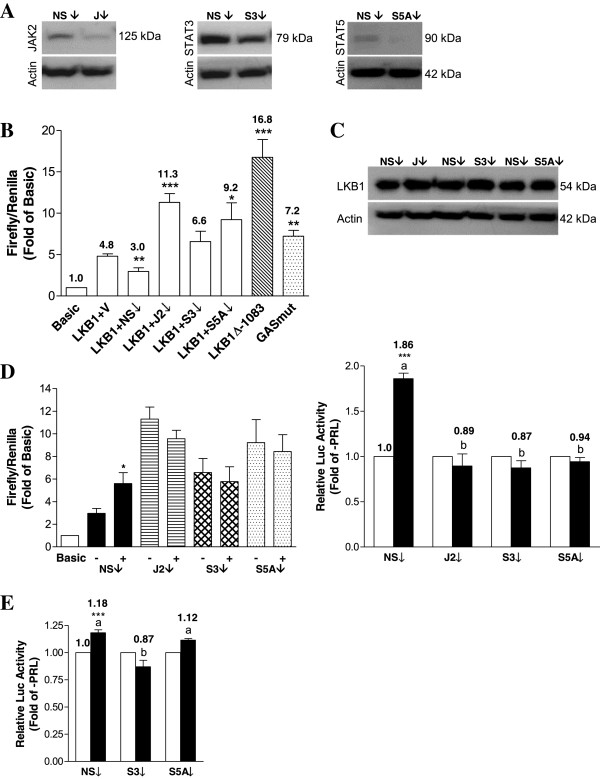
**JAK2, STAT3, and STAT5A differentially affect basal and PRL-stimulated LKB1 promoter activity in MDA-MB-231 cells.** MDA-MB-231 cells were transfected with non-specific siRNA (NS) or specific siRNAs targeting JAK2 (J2), STAT3 (S3), or STAT5A (S5A). **(A)** After 48 hr, knock-down was confirmed at the protein level by Western blotting. **(B)** Cells treated with siRNAs were co-transfected with Basic or LKB1 and pRL-TK, and lysates were assayed for dual luciferase activity. Data are presented relative to Basic. **(C)** Changes elicited by each siRNA at the basal transcriptional level were also assessed by examining total LKB1 protein levels. **(D)** Knock-down cells transfected with luciferase constructs as in **(B)** were cultured without or with 100 ng/mL of PRL for 24 hr, and lysates were analyzed using the dual luciferase assay. Changes in firefly/renilla relative to Basic are shown in the left panel, while the resulting fold changes in PRL-responsiveness are shown in the right panel (-PRL = open bars, +PRL = solid bars). **(E)** MCF-7 cells were transfected with the indicated siRNAs followed by transfection with the luciferase constructs. Results represent the mean of at least three independent experiments (±SEM). Different letters denote significant differences between the +PRL groups (p<0.05), and a star (*) indicates statistically significant increases in PRL-treated LKB1 promoter activity (p<0.05) compared to untreated NS siRNA.

To assess the contribution of the STAT pathway in MDA-MB-231 cells, we employed an siRNA approach. Transient knock-down of each target with a specific siRNA was first confirmed at the protein level compared to cells treated with a non-specific (NS) siRNA (Figure 5A). Transfection with JAK2 siRNA significantly up-regulated basal LKB1 promoter activity by approximately 3.8-fold relative to the NS control (p < 0.0001), an effect similar to that obtained using the LKB1Δ-1083 reporter construct (Figure 5B). Although knock-down of STAT3 increased basal promoter activity, the effect was not statistically significant (p = 0.08), while STAT5A knock-down significantly increased basal LKB1 promoter activity by approximately 3-fold (p < 0.05; Figure 5B). Decreasing the levels of either STAT3 or STAT5A using an siRNA approach resembled the effect observed with the GASmut reporter construct. Basal increases in LKB1 transcriptional activity were largely reflected at the protein level (Figure 5C). Knock-down of JAK2, STAT3, or STAT5A completely abolished the PRL-mediated induction of LKB1 promoter activity compared to the NS siRNA (Figure 5D). In MCF-7 cells, in which PRL treatment also increased LKB1 mRNA and protein levels (Figure 3A and B), the LKB1 promoter was mildly but significantly activated in response to treatment with PRL (by approximately 1.2-fold, p < 0.001), although not to the same level as observed in MDA-MB-231 cells (Figure 5E). Similar to MDA-MB-231 cells, knock-down of STAT3 in MCF-7 cells abolished PRL-responsiveness, although no effect was observed with the STAT5A siRNA (Figure 5E).

**Figure 6:**
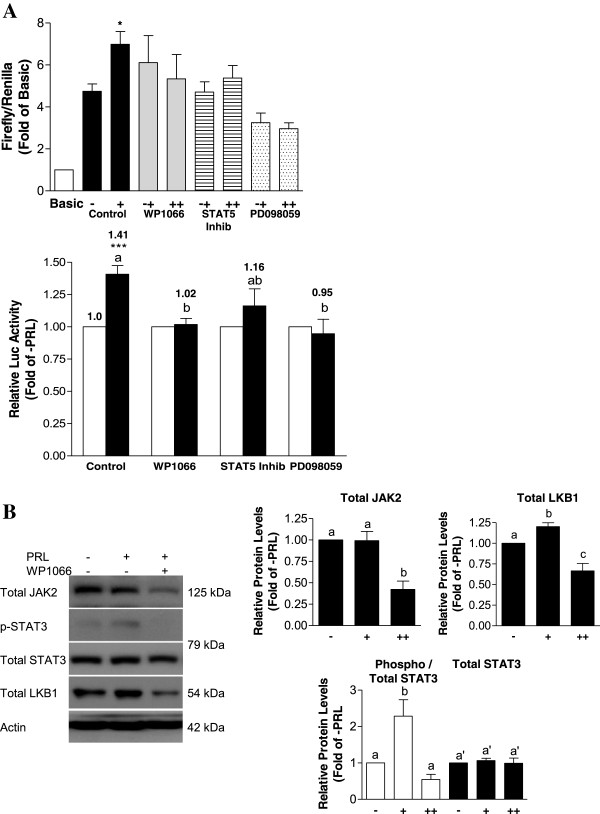
**WP1066, STAT5 inhibitor, and PD098059 affect PRL signaling to the LKB1 promoter in MDA-MB-231 cells. (A)** MDA-MB-231 cells were co-transfected with Basic or LKB1 and pRL-TK. Cells were cultured without (-, top panel; open bars, bottom panel) or with (+, top panel; solid bars, bottom panel) 100 ng/mL of PRL for 24 hr, and parallel groups of cells were pre-treated with WP1066, STAT5 inhibitor, or PD098059 for 2 hr prior to adding PRL for an additional 24 hr (++, top panel). Cell lysates were assayed for dual luciferase activity. Data in the top panel is presented relative to Basic, while the lower panel represents data normalized to the –PRL group. Results represent the mean of at least three independent experiments (±SEM), with different letters denoting significant differences between the PRL-treated groups (p<0.05) and a star (*) indicating statistically significant increases in PRL-treated LKB1 promoter activity (p<0.01) compared with the non-PRL-treated control. **(B)** A representative Western blot and densitometric analyses showing that the STAT3 pathway inhibitor WP1066 effectively degrades total JAK2 protein, blocks PRL-stimulated STAT3 phosphorylation, and reduces total levels of LKB1 protein.

Pretreatment of MDA-MB-231 cells with the STAT3 pathway inhibitor WP1066 significantly abolished PRL-mediated increases in promoter activity to levels comparable to the untreated control (Figure 6A). Although the STAT5 inhibitor did not significantly alter PRL-responsiveness compared to the untreated control, there was a trend toward reducing transcriptional activity mediated by PRL. PD098059, a MAPK pathway inhibitor, also completely abolished the effect of PRL (Figure 6A). WP1066 effectively blocked STAT3 phosphorylation induced by PRL after 24 hr, from a 2.3-fold increase to 0.54-fold (Figure 6B). Consistent with reports by others [44], it also degraded total JAK2 protein, as well as reducing the levels of total LKB1 (Figure 6B).

### PRL down-regulates LKB1 promoter activity in T47D human breast cancer cells

**Figure 7:**
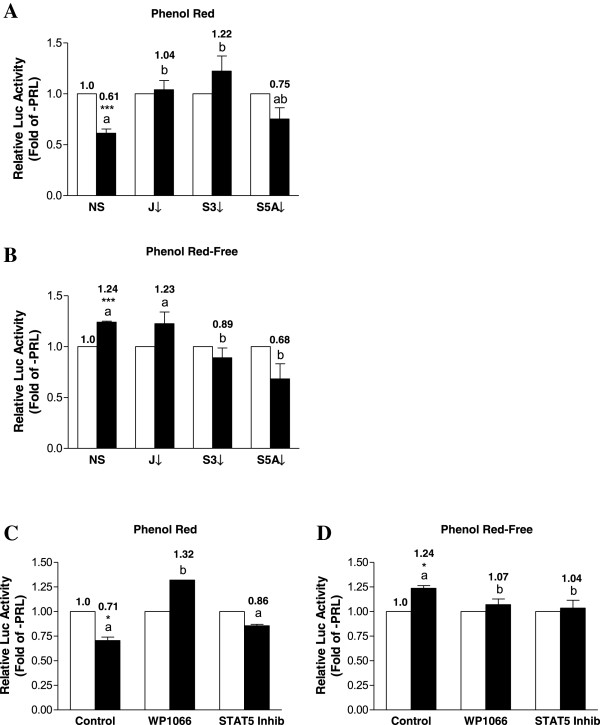
**Phenol red modulates PRL-responsiveness of the LKB1 promoter in T47D cells.** T47D cells were co-transfected with LKB1 and pRL-TK, followed by culture without (open bars) or with (solid bars) 100 ng/mL of PRL for 24 hr in **(A)** media containing phenol red or **(B)** phenol red-free media. Cells in **(A)** and **(B)** were first transfected with non-specific siRNA (NS) or specific siRNAs targeting JAK2 (J2), STAT3 (S3), or STAT5A (S5A) for 48 hr. Transfected T47D cells in **(C)** media with phenol red or **(D)** phenol red-free media were pretreated for 2 hr with WP1066 or the STAT5 inhibitor prior to adding PRL for an additional 24 hr. Lysates were assayed for dual luciferase activity. Data represent the mean of three independent experiments (±SEM) calculated relative to untreated controls, with different letters denoting significant differences between the PRL-treated groups and a star (*) indicating statistically significant increases in PRL-treated LKB1 promoter activity (p<0.05) compared with untreated controls.

Because T47D cells express high endogenous levels of the PRLR LF, but do not exhibit increases in LKB1 mRNA or protein following treatment with PRL, we evaluated the responsiveness of the LKB1 promoter to PRL in this breast cancer cell line. PRL induced the expected rapid activation of STAT5 (within 15 min, results not shown), and T47D cells were therefore treated with PRL for 15 min to assess the effect of knocking down JAK2, STAT3, and STAT5A on LKB1 transcriptional activity. Interestingly, PRL significantly down-regulated promoter activity in the NS siRNA control group by 40% (Figure 7A). In cells in which JAK2 or STAT3 were knocked down, PRL-induced promoter activity increased by approximately 1.7- or 2-fold in the presence of PRL (compare the results for NS at 0.61-fold to J↓ at 1.04-fold and S3↓ at 1.22-fold), while knock-down of STAT5A did not produce any significant changes (Figure 7A). These results are distinct from those observed using a similar siRNA approach in MDA-MB-231 or MCF-7 cells, which express low levels of PRLR LF. As we previously showed that EREs present in the promoter region may be important in regulating LKB1 expression in MCF-7 cells, and T47D cells are also ER-positive, we evaluated the effect of treating T47D cells with PRL under phenol red-free conditions. When the estrogen-like properties conferred by phenol red were withdrawn from the culture medium, treatment with PRL increased LKB1 promoter activity in a manner similar to what was observed in MDA-MB-231 cells (Figure 7B). Knock-down of STAT3 and STAT5A abolished PRL-responsiveness under these conditions (Figure 7B). Pretreatment with WP1066 or the STAT5 inhibitor produced results that were comparable to those obtained using siRNAs in either media containing phenol red or under phenol red-free culture conditions (Figures 7C and D, respectively).

### PRL induces binding of STATs to the GAS site in the distal LKB1 promoter region

**Figure 8:**
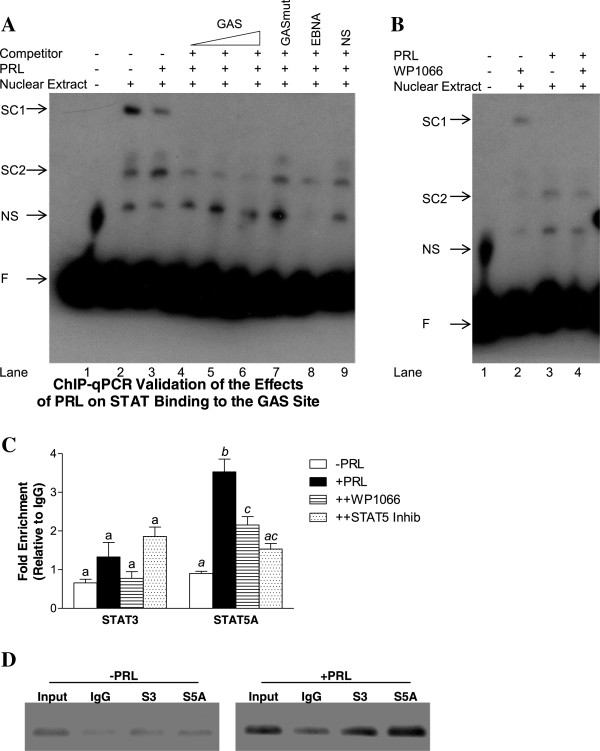
**PRL induces binding of STAT3 and STAT5A to the GAS site in the distal LKB1 promoter region. (A)** Nuclear extracts from MDA-MB-231 cells cultured for 24 hr without or with 100 ng/mL of PRL were added to binding reactions with a labeled LKB1 promoter probe spanning the GAS site and subjected to EMSA. Arrows indicated the formation of two specific complexes (SC1 and SC2), with PRL enhancing SC2 and decreasing SC1 (lanes 2 and 3). Nuclear extracts were pretreated with unlabeled GAS probe ranging from 1-4 pmol (lanes 4, 5, 6), unlabeled mutated GAS probe (GASmut) (lane 7), or unlabeled nonspecific (NS) probe (lane 9). **(B)** Nuclear extracts from cells pretreated with WP1066 for 2 hr prior to adding PRL for 24 hr were incubated with labeled probe, demonstrating reduced SC2 formation. Arrows indicate free probe (F) and a non-specific (NS) from probe alone. EMSAs in **(A)** and **(B)** represent results from at least two independent experiments. **(C)** and **(D)** represent ChIPs with anti-STAT3 and anti-STAT5A antibodies. A region spanning the putative GAS site in the distal LKB1 promoter region was PCR amplified from input, antibody-, or normal rabbit IgG-immunoprecipitated chromatin from untreated (-PRL) or treated (+100 ng/mL of PRL for 24 hr) MDA-MB-231 cells. **(C)** ChIP-quantitative real-time PCR validated the effects of PRL on STAT binding to the GAS site in the LKB1 promoter. STAT3 binding was reduced by WP1066, and PRL-enriched STAT5A binding was reduced by WP1066 and the STAT5 inhibitor. Results are expressed as fold enrichment relative to IgG normalized to a negative binding region. Different letters denote significant differences between treatment groups (p<0.05), representing results from two independent experiments. **(D)** ChIP PCR products analyzed by agarose gel electrophoresis confirmed the presence of one specific band at 184 bp enriched in the +PRL group.

To demonstrate that nuclear proteins present in MDA-MB-231 cells bind to the putative GAS site in the distal LKB1 promoter, EMSAs were carried out. Gel shift experiments revealed the formation of specific complexes in the presence of the GAS probe (Figure 8A). Nuclear extracts isolated from cells treated with PRL for 24 hr showed that specific complex 1 was reduced while complex 2 increased compared to complexes formed in extracts derived from untreated cells (Figure 8A). An unlabeled GAS probe effectively competed with formation of complex 2, while unlabeled oligonucleotides containing either a mutated GAS sequence or an unrelated nonspecific probe sequence were unable to compete for complex formation. Pretreatment with WP1066 prior to stimulation with PRL reduced formation of complex 2 (Figure 8B).To definitively demonstrate that PRL increased the binding of STAT3 and/or STAT5A to the GAS site, ChIP assays linked with quantitative real time PCR were carried out on chromatin isolated from unstimulated and PRL-stimulated MDA-MB-231 cells. Quantitatively, the significant 4-fold enrichment of STAT5A binding to the LKB1 promoter region containing the GAS site in response to PRL treatment was significantly reduced by pretreating cells with WP1066 or the STAT5 inhibitor (Figure 8C). Although not statistically significant, STAT3 binding at this site was also increased by PRL by approximately 2-fold, an effect that was abrogated by pretreatment with WP1066 but not the STAT5 inhibitor (Figure 8C). Gel eletrophoresis of the real-time PCR reactions visually showed that, compared to IgG, STAT3 and STAT5A binding was higher following PRL treatment (Figure 8D).

## Discussion

Current research suggests that loss of LKB1, an important multi-tasking protein, is linked with changes in cell polarity and cytoskeletal rearrangements, and that these changes may drive tumor growth when the cellular metabolic balance is disrupted in response to energetically unfavorable conditions. We previously showed that activation of the AMPK pathway involves LKB1 in human breast cancer cells. In the current investigation, we suggest that LKB1 may also control specific structural changes that could potentially be important during disease progression, as its knockdown in MDA-MB-231 cells produced marked morphological changes, warranting further investigation into the mechanisms that control its expression in breast cancer cells. Similar to results observed in our study following knock-down of LKB1, knock-down of WNT in MDA-MB-231 cells altered their morphology, indicated by loss of the typical spindle shape, with cells becoming rounded [45]. LKB1 has been linked with the WNT pathway (reviewed in [46]), and assays carried out in Xenopus and mammalian cells demonstrate that LKB1 upregulates β-catenin only in the presence of WNT [47]. Furthermore, in Peutz-Jeghers syndrome polyps, the expression of LKB1 and β-catenin were positively correlated [48]. We report that knock-down of LKB1 in MDA-MB-231 cells is associated with decreased levels of β-catenin and β-tubulin, a key component of microtubules. In mice, knockdown of *Lkb1* results in disintegration of neurofilaments and microtubules in the spinal cord, with decreased staining for β-tubulin III [49], and loss of pancreatic Lkb1 deregulates AMPK and protein family members that establish tight junctions and mediate tubulin dynamics, leading to acinar polarity defects and cystic neoplasms [50]. Furthermore, in another study identifying LKB1 as a critical mediator in the WNT pathway, microtubules were affected in *Lkb1* knockout cells undergoing excessive cilia disassembly [51]. Loss of polarity and cytoskeletal rearrangements are generally associated with tumor progression, and these changes are linked with the epithelial-to-mesenchymal transition. Altered levels of LKB1 could change expression of β-catenin and other key markers of this process, thereby driving asymmetric cell division and shifting the balance between self-renewal, differentiation, and de-differentiation [52]. Others have shown that by activating JAK2 in MDA-MB-231 cells, PRL regulates the morphogenic program, suppressing metastatic potential and acting as an invasion suppressor [53], and long-term administration of PRL to cultured neonatal rat pancreatic islet cells increases β-catenin levels [54]. While the molecular basis underlying how LKB1 affects cell polarity and cytoskeletal arrangements in breast cancer cells remains to be determined, our study focused on gaining a better understanding of how LKB1 expression is regulated, which may vary depending on the molecular signature of different breast cancer cells.

We previously reported that LKB1 protein levels increase in response to PRL in MDA-MB-231 cells [26], suggesting that LKB1 expression could be transcriptionally regulated. While variable levels of LKB1 have been reported in MDA-MB-231 cells [55,56], a recent study corroborates our finding that LKB1 is present and functional in this particular human breast cancer cell line [57]. These cells are commonly used in experimental models to represent aggressive, basal-like, triple-negative human breast cancer cells. To determine whether PRL could directly alter LKB1 expression, we examined the PRLR status in MDA-MB-231 cells, as well as several other cell lines. Seventy to 95% of human breast cancers express the PRLR [58,59]. It has been suggested that, compared to MCF-7 cells, the PRLR is not expressed in MDA-MB-231 cells due to DNA hypermethylation of its promoter region [60], although expression at the protein level was not assessed. Others have shown that several isoforms of PRLR, including the LF, SF1a, and SF1b, are expressed at the protein level in both MCF-7 and MDA-MB-231 xenografts [37]. Furthermore, changes in the expression of several different homo- and heterodimeric PRLR pairs consisting of the long and short forms were observed in MDA-MB-231 cells over the course of prolonged PRL stimulation [61]. Activation of JAK2 and signaling to STATs has been reported for the LF, as well as several other splice variants (reviewed in [62]). In the current investigation, we show that PRLR LF, and potentially several other isoforms that also support signaling through STATs, are expressed in MDA-MB-231 cells, and that JAK2 and STAT3, as well as STAT5, are activated following sustained PRL treatment.

PRL has been shown to up-regulate the transcription of numerous target genes by promoting signaling to GAS sites that are bound by STAT proteins, including cyclin D1 [32,33] and β-casein [30,31]. The activity of a LKB1 promoter-luciferase reporter construct was significantly enhanced by PRL in MDA-MB-231 cells, an effect that was lost upon truncation of the distal promoter region containing a putative GAS/STAT binding site. This GAS site was confirmed to be important in mediating transcriptional activity, and JAK2, STAT3, and STAT5A were shown to be required for PRL to stimulate the LKB1 promoter in MDA-MB-231 cells. Furthermore, *in vivo* binding of STAT3 and STAT5A to the GAS site was enriched in MDA-MB-231 cells following treatment with PRL. The contribution of STAT5A in regulating PRL-responsiveness was unexpected, given that STAT5 phosphorylation was very low in this cell line. Its importance was, however, definitive, as both chemical and siRNA-mediated inhibition blocked PRL-responsiveness of the LKB1 promoter. The effect of PRL on STAT activation was not observed until 24 hours post-stimulation. A similar time frame has been described for assessing STAT5A-mediated reporter gene activity of other promoters in breast cancer cells stimulated with a similar concentration of PRL [63]. However, it is possible that sustained treatment with PRL activates other proteins first, particularly given the low levels of PRLR LF in MDA-MB-231 cells. These proteins could potentially induce the synthesis of factors that in turn activate JAK/STAT signaling, thereby indirectly contributing to LKB1 transcriptional activity. It is possible, for example, that the action of phosphatases is inhibited, the effects of which would accumulate over time. Indeed, others have shown that levels of the JAK2 phosphatase, PTP1B, are inversely correlated with nuclear levels of phosphorylated STAT5A and B in human breast cancer and that PTP1B suppressed the levels of PRL-induced phosphorylated STAT5A [64]. The lack of STAT5 phosphorylation in the presence of continued total STAT5 protein expression in clinical breast cancer samples suggests that tyrosine phosphatases are important regulators, and Johnson et al. (2010) show that PTP1B protein levels may be higher in MCF-7 and MDA-MB-231 cells compared to T47D cells [64]. Our results indicate that total levels of STAT5 are relatively abundant in MDA-MB-231 cells, and changes in PTP1B levels may therefore be of relevance to our study. We aim to investigate the mechanism(s) underlying the delayed response reported in the current investigation in future studies. Nevertheless, it is clear that STAT3 and STAT5 both play a role in regulating LKB1, and that PRL and other cytokines known to induce STAT signaling, such as IL-6, modulate its expression in a cell type-dependent manner. Interestingly, PRL has been shown to induce the production of IL-6 in murine dendritic cells *in vitro* and *in vivo*[65], and MDA-MB-231 cells have been shown to secrete IL-6 *in vitro*[66]. It is therefore possible that the longer time frame required for PRL to activate JAK/STAT3 and elicit its effect on LKB1 in MDA-MB-231 cells may require up-regulated production of IL-6, which, via signaling through the IL-6 receptor composed of IL6Rα and GP130 heterodimers, then stimulates the LKB1 promoter through autocrine activation of the JAK/STAT pathway. STAT5 is phosphorylated in endothelial cells treated with IL-3, which suggests an involvement in angiogenesis and cell motility [67], and it is therefore also possible that IL-3 may play a role in breast cancer cells. It will be of considerable interest to explore whether PRL induces IL-6 or IL-3 expression in MDA-MB-231 cells, and whether depleting these cytokines from conditioned media or blocking their receptors affects LKB1 expression.

Truncation of the region spanning -1889 to -1083 dramatically increased basal transcriptional activity, while mutation of the GAS site only mildly lifted basal repression, suggesting that (an)other site(s) within these 800 base pairs likely confers the major inhibitory effect. Knockdown of STAT3 and STAT5, similar to GAS mutation, did not lift basal repression to the same extent as promoter truncation. In contrast, knockdown of JAK2 produced a dramatic effect similar to truncation, suggesting that broader JAK2-mediated signaling contributes to basal transcriptional repression at the LKB1 locus. While knockdown of one STAT family member could potentially lead to a compensatory action by other family members, it is also possible that STATs, in particular STAT5A, are not repressive on their own, but interact with or enhance the action of (an)other repressor(s) in the absence of PRL. For example, in the case of cyclin D1, PRL stimulation decreased constitutive binding of OCT-1 to a specific site in the promoter region, thereby lifting basal transcriptional repression, and PRL-mediated cyclin D1 promoter activity increased in response to JAK2/STAT5 signaling involving an adjacent GAS site [33]. Interestingly, we identified two putative OCT-1 sites in close proximity to the GAS site within the distal LKB1 promoter, and this potential mechanism of regulating basal LKB1 transcription will be explored in future studies, particularly given that EMSAs indicated the presence of a specific complex that is reduced when cells are treated with PRL.

PRL may potentially promote synergism or induce antagonism between STATs and other signaling components. In particular, contributions through the MAPK pathway cannot be discounted, given that a putative AP-1 site also maps to the distal LKB1 promoter region. PRL has been shown, in various cell types, to activate JNK, p38 MAPK, and ERK1/2, thereby inducing DNA binding at AP-1 sites (reviewed in [32]), and PRL RAS-dependently modifies the composition and activity of complexes at a distal AP-1 site in the cyclin D1 promoter [68]. In addition to JAK-mediated signaling, activation of the RAS-MAPK pathway leads to the specific phosphorylation of a serine near the C-terminus of most STATs, and, while not required for STAT activity, this change may enhance STAT-mediated transcriptional activation [69]. We found that PD098059, a specific MEK1/2 inhibitor, repressed both basal and PRL-stimulated LKB1 promoter activity. In addition, a putative early growth response 1 (EGR-1) binding site is also present in the LKB1 promoter, and it has been shown that PRL stimulates expression of vascular endothelial growth factor (VEGF) via Egr-1 in a JAK2 and MAPK-dependent manner in murine mammary epithelial cells [70]. Another interesting putative site mapping to the distal LKB1 promoter is a HIF1α binding motif. HIF1α, together with STAT3, has been implicated in transcriptionally regulating VEGF expression via SRC in pancreatic and prostrate carcinomas [71], suppression of HIF1α and STAT3 is associated with anti-angiogenic activity in hypoxic prostate cancer cells [72], and PRL increases VEGF expression in bovine mammary cells [73]. Of note, LKB1 is required for angiogenesis in endothelial cells [74], and it is therefore possible that STATs and HIF1α together control the transcriptional activity of LKB1 in breast cancer cells under certain conditions.

Similar to MDA-MB-231 cells, truncating the distal LKB1 promoter region containing the putative GAS site in T47D cells increased basal transcriptional activity. In the presence of phenol red, which has estrogenic properties [75], PRL down-regulated LKB1 promoter activity in T47D cells, reciprocal to its action in MDA-MB-231 cells. Blocking signaling through STAT3, but not STAT5A, reversed this effect, as did culture of T47D cells in phenol red-free conditions. In the absence of phenol red, LKB1 promoter activity in response to PRL was also affected by STAT3. These findings suggest that up-regulation of LKB1 transcriptional activity by PRL is cell type-dependent, and may be influenced by estrogen, as well as STAT3, in ER-positive breast cancer cells. PRL increases ERα expression in the ovary [76], and this could potentially be a mechanism that down-regulates LKB1 transcriptional activity in T47D cells in our study. Nuclear receptors, including ER, are negative modulators of STAT3 in multiple myeloma cells [77]. Activation of STAT3 by IL-6 and subsequent changes in target gene expression are suppressed by 17β-estradiol in MCF-7 cells, an effect attributed to the direct interaction between ER and STAT3 that prevents the DNA binding activity of STAT3 [78]. Consistent with the findings in T47D cells reported here, we and others have previously shown that LKB1 expression may be transcriptionally altered by 17β-estradiol in MCF-7 cells [29,79], and while PRL does increase LKB1 promoter activity in MCF-7 cells, the effect is significantly blunted compared to MDA-MB-231 cells. There appears to be a mechanistic relationship between PRL, ERα, and STAT3 in regulating LKB1 expression, the details of which remain to be determined.

Cancer cells commonly develop resistance to therapies over the course of treatment, and it is therefore advantageous to simultaneously target several signaling pathways to provide effective therapeutic intervention. Recently, it has been shown that methylsulfonylmethane (MSM), a natural compound without any known toxicities, effectively inhibits the STAT3/VEGF and STAT5B/insulin-like growth factor receptor (IGF-1R) pathways in human breast cancer cells [80]. A proposed mechanism driving MSM action in MDA-MB-231 cells is its prevention of STAT binding to sites within target gene promoters [80]. We have not examined the contribution of STAT5B in our study, although it has been suggested that the balance between STAT5A and B expression may be important in breast cancer progression [81]. A recent report has suggested therapeutically targeting phosphoinositide 3 kinase (PI3K)/mTOR signaling in conjunction with suppression of JAK2/STAT5 in certain triple-negative breast cancers [82]. Treatment of triple-negative breast tumors with PI3K inhibitors resulted in upregulation of the JAK2/STAT5 pathway, leading to increased rates of metastasis, but when mice were treated with drugs that blocked both PI3K and JAK2/STAT5, tumor cells proliferated more slowly and metastasized less readily, and the survival rate of the animals increased [82]. Activated Stat5 has also been shown to increase metastases of prostate cancer cells in nude mice, promoting migration and invasion, also inducing rearrangements of the microtubule network [83]. The importance of targeting more than one pathway, or more than one STAT protein, is underscored by the finding that STAT3 suppresses the transcription of proapoptotic genes in breast cancer cells [84]. Feedback may also play a role, as loss of STAT5A using SRC inhibitors facilitates the recovery of STAT3-mediated signaling, thereby improving cell survival in head and neck squamous carcinomas [85].

## Conclusions

Understanding how PRL and other extracellular stimuli signal to key sites in the LKB1 promoter will provide important insight into the cellular responses that change during breast cancer progression. Other factors of interest are cytokines, particularly IL-6, which plays a role in epithelial tumors and is linked with differential STAT3 signaling [86]. A mechanistic approach is relevant, given that LKB1 acts either as an inducer or suppressor of apoptosis in a cell-type dependent manner that is linked with the severity of energy stress [23-25], and activation of the LKB1-AMPK pathway decreases ATP-consuming processes while increasing ATP production, which fits well with the energy-compromised status of aggressive cancer cells. Upregulation of LKB1 may provide a means for cancer cells to survive under energetically unfavorable conditions, and hormones/cytokines may differentially alter their metastatic potential due to cytoskeletal changes linked to LKB1. It is becoming apparent that breast cancer therapies need to be “tailored” to the individual patient in a manner dependent on the unique characteristics of the originating cancer cells. Examining the contribution of STAT proteins in regulating key cellular proteins like LKB1, and their relationship with different levels of hormone-responsiveness, is an integral component of this process.

## Competing interests

The authors declare that they have no competing interests.

## Authors’ contributions

KL-M conceived and designed the study, conducted all experiments, performed statistical analyses, prepared figures, and drafted the manuscript. GS provided funding and critically reviewed the manuscript. Both authors have read and approved the final manuscript.

## Authors’ information

Katja Linher-Melville supported by a Canadian Breast Cancer Foundation (CBCF) fellowship.

Gurmit Singh research supported by an operating grant from Canadian Institutes for Health Research (CIHR).

## Pre-publication history

The pre-publication history for this paper can be accessed here:

http://www.biomedcentral.com/1471-2407/14/415/prepub
